# Meta-Analysis of Rose Rosette Disease-Resistant Quantitative Trait Loci and a Search for Candidate Genes

**DOI:** 10.3390/pathogens12040575

**Published:** 2023-04-08

**Authors:** Tessa Hochhaus, Jeekin Lau, Cristiane H. Taniguti, Ellen L. Young, David H. Byrne, Oscar Riera-Lizarazu

**Affiliations:** Department of Horticultural Sciences, Texas A&M University, College Station, TX 77843-2133, USA

**Keywords:** QTL, virus resistance, meta-analysis, Rosa, rose rosette virus

## Abstract

Rose rosette disease (RRD), caused by the rose rosette emaravirus (RRV), is a major viral disease in roses (*Rosa* sp.) that threatens the rose industry. Recent studies have revealed quantitative trait loci (QTL) for reduced susceptibility to RRD in the linkage groups (LGs) 1, 5, 6, and 7 in tetraploid populations and the LGs 1, 3, 5, and 6 in diploid populations. In this study, we seek to better localize and understand the relationship between QTL identified in both diploid and tetraploid populations. We do so by remapping the populations found in these studies and performing a meta-analysis. This analysis reveals that the peaks and intervals for QTL using diploid and tetraploid populations co-localized on LG 1, suggesting that these are the same QTL. The same was seen on LG 3. Three meta-QTL were identified on LG 5, and two were discovered on LG 6. The meta-QTL on LG 1, MetaRRD1.1, had a confidence interval (CI) of 10.53 cM. On LG 3, MetaRRD3.1 had a CI of 5.94 cM. MetaRRD5.1 had a CI of 17.37 cM, MetaRRD5.2 had a CI of 4.33 cM, and MetaRRD5.3 had a CI of 21.95 cM. For LG 6, MetaRRD6.1 and MetaRRD6.2 had CIs of 9.81 and 8.81 cM, respectively. The analysis also led to the identification of potential disease resistance genes, with a primary interest in genes localized in meta-QTL intervals on LG 5 as this LG was found to explain the greatest proportion of phenotypic variance for RRD resistance. The results from this study may be used in the design of more robust marker-based selection tools to track and use a given QTL in a plant breeding context.

## 1. Introduction

The economic importance of roses (*Rosa* L. sp.), as one of the most popular cut flowers and ornamental plants, is estimated at USD 28 billion globally [1]. Unfortunately, roses are vulnerable to diseases such as black spot, cercospora leaf spot, downy and powdery mildew, and rust, which threaten roses’ value as a landscape crop. Rose rosette disease (RRD) is the most important viral disease of roses in the USA [2]. RRD is caused by the rose rosette emaravirus (RRV), which is spread by an eriophyid mite, *Phyllocoptes fructiphilus* Keifer. The most common control is the removal and destruction of infected plants. Management practices include exclusion of the virus or controlling the mite to limit movement [3]. Chemical control of the mite is generally ineffective at controlling the virus due to the short inoculation access period [4]. The disease can be fatal and shows symptoms of elongated shoots, known as witches’ broom, rosetting, leaf distortion, red or yellow leaves, excessive prickles, and increased susceptibility to other stresses or diseases. Plant death is expected 3–5 years after the initial infection [4,5]. Due to the high disease pressure in some areas and the lack of control methods, finding naturally resistant materials is essential.

This disease has become more prevalent and causes significant losses commercially in production and publicly in rose plantings. Therefore, research efforts to discover rose cultivars and species that display RRD resistance have become more extensive in the past couple of decades. This requires multiyear trials to determine the level of resistance [6]. Although the level of susceptibility to RRD varies, no complete resistance to RRD has been found among the major commercial rose cultivars. However, high resistance has been found among a few cultivars with parentage originating from various resistant North American (*R. acicularis*, *R. arkansana*, *R. blanda*, *R. californica*, *R. carolina*, *R. palustris*, *R. pisocarpa*, and *R. setigera*) and Asian species (*R. spinossisima*, *R. wichurana*, and *R. bracteata*). Most of these species and species hybrids have not been significantly explored for commercial breeding [7].

Plants have evolved to protect themselves when invaded by pathogens. For viruses specifically, mechanisms include RNA silencing, innate immunity, ubiquitination–mediation, and translational repression as a few of the major defenses [8]. Plant hormones are also key in defense against pathogens. Salicylic acid, jasmonic acid, and ethylene alter a plant’s response against biotic stresses. Hormones related to plant growth and development (auxin, brassinosteroids, cytokinins, and abscisic acid) are also involved in plant–pathogen interactions [9]. Chaperonins have also been found to be involved in the regulation of virus reproduction and movement [10]. They can be components of R-mediated resistance [9], which produces a hypersensitive response to prevent the spread of the infection.

Although QTL for resistance to some emaraviruses in species other than roses have been described [11,12,13,14], R genes, per se, have not been identified. Two recent studies, one on inter-related diploid populations by Young et al. [6] and another on two interconnected tetraploid populations by Lau et al. [15], identified multiple quantitative trait loci (QTL) related to RRD partial resistance. Both studies performed QTL analysis on disease severity and virus RT-qPCR Ct values [16]. Lau et al. identified QTL on linkage groups (LGs) 5, 6, and 7, accounting for 18–40% of the phenotypic variance, with the greatest effect for resistance attributed to LG 5 [15]. By performing a joint QTL analysis of two interconnected tetraploid populations using diaQTL [17], an additional QTL on LG 1 for Ct value was found. Young et al. found QTL on LGs 1, 3, 5, and 6, with the QTL on LG 5 being the most consistent, accounting for approximately 20–40% of the phenotypic variance [6].

QTL mapping can be influenced by many factors, such as population size, the germplasm base, environmental conditions, and the number and density of genetic markers [18,19]. Using a meta-analysis, one identifies consistent QTL with major effects called meta-QTL. The objective of a meta-analysis is multifaceted. It increases QTL detection power, increases sample size, and uses data and information between studies, thereby gaining insight into the genetic architecture of the trait of interest [20]. In addition, meta-QTL with smaller and better-defined confidence intervals and consistent effects across populations are useful for marker-assisted selection [19]. This method has been widely used in a variety of disciplines, including medical, social, and behavioral sciences [20], and within horticulture and agriculture on a multitude of crops [19,21,22].

In this study, we conducted a meta-analysis to combine the diploid [6] and tetraploid [15] datasets to understand the relationship between RRD-resistant QTL in these two germplasm groups. In addition to using the data directly from these studies, we remapped all populations to improve the marker order and genetic distance estimation in both the diploid and tetraploid linkage maps to better define RRD-resistant QTL intervals and focus our candidate gene search within these RRD meta-QTL regions.

## 2. Materials and Methods

This meta-analysis is largely based on two studies described by Young et al. [6] and Lau et al. [15]. Additionally, we remapped marker data from the pertinent populations to improve both the diploid and tetraploid linkage maps to better define RRD-resistant QTL intervals and use these refinements to focus our candidate gene searches.

### 2.1. Original Studies

#### 2.1.1. Mapping Populations

Lau et al. [15] utilized two tetraploid *Rosa* L. F_1_ populations with the common parent, Brite Eyes (‘RADbrite’, Brite Eyes^TM^); the two populations are crosses between Stormy Weather (‘ORAantanov’, Stormy Weather^TM^) × Brite Eyes (SW × BE) and Brite Eyes × My Girl (‘BAIgirl’, Easy Elegance^®^ My Girl) (BE × MG), with a population size of 200 and 157, respectively. Concerning rose rosette disease resistance, Brite Eyes, My Girl, and Stormy Weather show moderate to high susceptibility.

Young et al. [6] used a multi-parent inter-related diploid population named TX2WSE. ‘Papa Hemeray’ (PH), ‘Srdce Europy’ (SE), Swamp Rose, and *R. setigera* ARE, possible sources of RRD resistance, were crossed with Lena (‘BAIlena’), Ole (‘BAIole’), and the Texas A&M breeding lines M4-4, TAMU7-20, and TAMU7-30. From these crosses, eight populations were produced with a total of 382 progeny, 248 of which were phenotyped for RRD.

Young et al. [6] and Lau et al. [15] evaluated diploid and tetraploid populations for RRD at the University of Tennessee AgResearch Plateau Research and Education Center in Crossville, TN, in a randomized complete block design. RRD infection was augmented through proximity of infected wild roses as well as the attachment of infected rosettes, carrying vector mites, to healthy plants two to three times per year. Phenotyping for RRD infection was performed on a 0–3 scale for disease severity (0 = no symptoms, 1 = single shoot with a rosette, 2 = 2–3 shoots with a rosette, and 3 = 4 or more shoots with a rosette). Evaluations were performed in 2019, 2020, and 2021. Populations studied by Lau et al. [15] were phenotyped twice in 2021, with the second evaluation of that year (2021b) being on a scale of 0–5, which was later scaled to match the 0–3 scale. In populations studied by Young et al. [6], RRD was evaluated once in 2021, using the 0–5 scale, and rescaled to match the other years. Testing for virus presence was performed using RT-qPCR. Plants were repeatedly screened until a plant tested positive. For both studies, the lowest resulting cycle threshold (Ct), corresponding to the highest virus content, was used for QTL mapping.

#### 2.1.2. Linkage Mapping

Young et al. [6] used the three largest diploid populations for linkage mapping: (1) J06-20-14-3 × Papa Hemeray (J14.3 × PH), (2) TAMU7-20 × Srdce Europy (T7.20 × SE), and (3) TAMU7-30 × Srdce Europy (T7.30 × SE). SNP markers, from genotyping by sequencing (GBS), were filtered to eliminate markers of low quality: more than 10% missing data, markers that did not align to the reference genome, missing parental genotypes, and multiallelic or a high proportion of deletion alleles. Young et al. [6] constructed linkage maps for each population using polymapR [23], and markers were ordered using MDSMap as implemented in polymapR [24]. LPMerge [25] implemented in the R package mapfuser [26] was used to construct the consensus map, which had a total of 2677 markers and a total length of 758.2 cM.

In Lau et al. [15], polymapR [24] was also used. SNP markers, genotyped on the Axiom WAgRhSNP 68K array, were filtered so that only markers with less than 1% missing data for markers and individuals were used. Marker ordering was produced using MDSMap and then BLASTed against the genome to obtain marker positions. Genotype and homolog probabilities were generated by Lau et al. [15] using MAPpoly [27]. The map from the Brite Eyes × My Girl population had a map length of 542.67 cM and 5675 markers, while the map from the Stormy Weather × Brite Eyes had a map length of 541.56 cM and 5494 markers.

#### 2.1.3. QTL Mapping

Young et al. [6] utilized FlexQTL^TM^ [28] for the QTL analysis. FlexQTL^TM^ utilizes a Bayesian framework, which is designed to use inter-related populations to follow alleles of interest to the founder (identity by descent (IBD)). The analysis was run four times for each trait for QTL reproducibility. A 2lnBF greater than two was considered positive evidence of a QTL. Haplotyping was performed on FlexQTL^TM^ and the R package PediHaplotyper [29]. QTL discovered by Young et al. [6] are presented in Table 1.

Lau et al. [15] used genotype probabilities from MAPpoly [27] for QTL scans in QTLpoly [30]. QTL discovered by Lau et al. [15] in the two populations are presented in Table 2. PolyOrigin [31] was used to reconstruct phased haplotypes in all progeny, which was then utilized for a joint-family QTL analysis in diaQTL [17]. QTL discovered by Lau et al. [15] in the joint analysis are presented in Table 3.

### 2.2. Remapping Based on Marker Physical Order

To further narrow the metaQTL regions and focus our candidate gene scan, we remapped all populations in the same framework. We used the three largest populations from the diploid study [6] (T7.20 × SE, T7.30 × SE, and J14.3 × PH) and the two tetraploid populations from Lau et al. [15] (SW × BE, BE × MG). Previous diploid and tetraploid maps were mapped in polymapR [24] using an MDS mapping approach, which results in many small local inversions. In the remapping, we grouped and ordered the markers by their physical position aligned to the *Rosa chinensis* genome v1.0 [32]. Markers positioned in close physical order proximity but with high recombination fractions with adjacent markers were filtered out. Once the groups and orders were established, the genetic distances were estimated for each map using the hidden Markov model (HMM) multi-point approach implemented in MAPpoly [27]. A global error rate of 5% was set in the HMM emission function to fix potential genotyping errors according to the multi-point information. The haplotype genotypes probabilities were generated and then used in QTLpoly [30] to scan for QTL. QTL discovered in the diploid and tetraploid populations are found in Table 4 and Table 5. Marker names and positions were extracted and used in Biomercator v4.2.3 [33,34] input file construction. Map summary information can be found in Supplementary Tables S1–S5.

### 2.3. Input File Construction

Two input files for each population (map file and QTL file) were entered in Biomercator v4.2.3 [33,34]. The QTL file included QTL name, year, location, linkage group, chromosome position (cM), confidence interval (cM), proportion of the phenotypic variance explained (PVE%), and LOP score (−log(*p*-value)) [28] or equivalent (Bayes factor [28] or ΔDIC [17]). Marker files were created for each population containing general population information, such as population size, genus and species, and cross-type, as well as map information, including mapping unit and quality. The map files contained lists of markers and marker positions in centiMorgans (cM) per chromosome and LG.

Due to the difference in genotyping platforms between the two studies, marker connectivity was derived via the markers’ physical locations. All markers within a 5 kilobase pairs (Kbp) window were considered to represent the same locus and were placed in that bin, and the locus was given a new identifier. This allowed the connection of markers from maps of the various datasets.

The map and QTL files for each of the populations, (1) the TX2WSE multi-parent diploid population, (2) SW × BE tetraploid population, (3) BE × MG tetraploid population, (4) joint tetraploid population (SW × BE × MG), (5) remapped T7.20 × SE, (6) remapped T7.30 × SE, (7) remapped SW × BE, and (8) remapped BE × MG, were uploaded to Biomercator for meta-analysis. J14.3 × PH was not included as no significant QTL were discovered.

### 2.4. Meta Analysis

Biomercator has an iterative map function, with which we created a consensus map using the remapped BExMG population as the reference (Figure S1). We chose this population as it is the largest map and contains markers in physical order. QTL from each population were then projected onto this map using the QTLProj function. To analyze meta-QTL, we used the Veyrieras method [35], which is an extension of the Goffinet and Gerber method [36]. The Veyrieras method implements a statistical framework to combine distinct genetic maps as well as a clustering approach to decide how many meta-QTL underly the collection of QTL produced by various studies [35]. LGs 1, 3, 5, and 6 with RRD-resistant QTL from both the diploid and tetraploid populations were included in the meta-analysis. The procedure has two steps. First, ClustQTL produces three files: a summary of the clustering for each linkage group, a summary of the values of the model choice criteria, and a file that gives the optimum QTL number. These files are then used in the second step using the MQTLView function, which produces a visual representation of the meta-QTLs in a new map. The best model for each LG was selected based on the lowest Akaike information criterion value (AIC). Positions and intervals were generated for each consensus QTL (meta-QTL), and these were given the name, MetaRRD.

### 2.5. Candidate Gene Scan

The physical size of meta-QTL intervals in mega base pairs (Mbp) was estimated by extracting the markers used in the projection of meta-QTL in Biomercator. The markers closest to the meta-QTL start and end positions were used to determine the Mbp positions using the *Rosa chinensis* whole genome v1.0 as a reference [32]. Genes localized in intervals discovered in the meta-analysis were extracted using the Tripal MegaSearch in the Genome Database of Rosaceae (GDR) at rosaceae.org [37]. This was performed by searching for genes/transcripts data types in the *R. chinensis* whole genome v1.0 assembly and annotation corresponding to intervals of our meta-QTL. Genes were manually searched for those encoding proteins with a nucleotide-binding site and leucine-rich repeats (NBS-LRRs) and proteins with a central nucleotide-binding domain, known as NB-ARCs, as well as genes related to anti-viral mechanisms, defined as genes that either hinder the reproduction or movement of the virus and its products. We also searched for genes related to general defense response.

## 3. Results

### 3.1. Meta-Analysis

**Figure 1:**
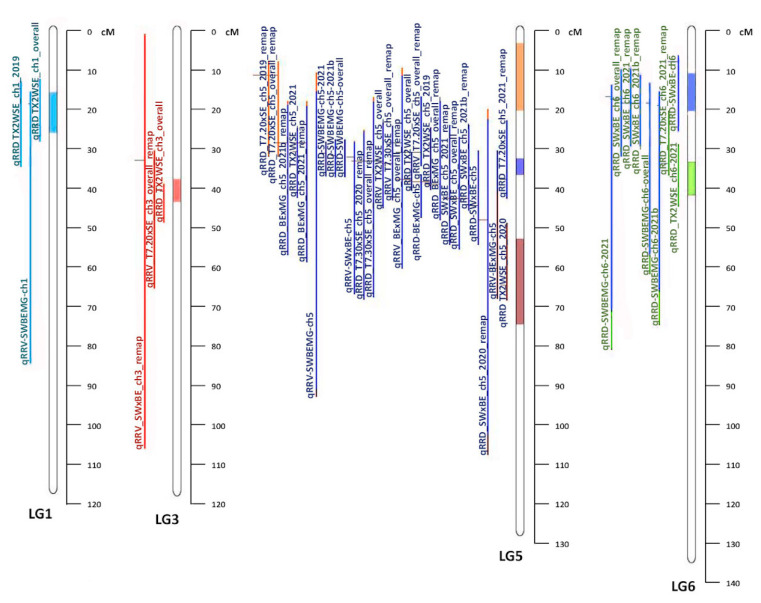
Representation of LGs 1, 5, and 6 for individual QTL from the two studies and the meta-QTL associated with rose rosette disease resistance in roses. The name of the individual QTL is on the left, and the meta-QTL regions are depicted on each LG.

LGs 1, 3, 5, and 6 were included in the meta-analysis as they had QTL from both the diploid and tetraploid populations. QTL from each population were projected onto the consensus map using the QTLProj function in Biomercator with a total of three QTL on LG 1, three QTL on LG 3, twenty-eight QTL on LG 5, and eight QTL on LG 6. The best model for the number of meta-QTL per LG was based on the lowest AIC. One meta-QTL was found on LG 1, one meta-QTL on LG 3, three on LG 5, and two meta-QTL were identified on LG 6. The position of meta-QTL in relation to the input QTL from the individual populations can be seen in Figure 1. On LG 1, the meta-QTL, named MetaRRD1.1, had a confidence interval (CI) of 10.53 cM. The average CI of individual QTL intervals from both studies was 35.75 cM, which was approximately three times as large as the CI for MetaRRD1.1. MetaRRD3.1 had a CI of 5.94 cM, making it more than four times smaller than the average of the QTL contributing to it (26.87 cM). For LG 5, the first meta-QTL, MetaRRD5.1, had a CI of 17.37cM; the second, MetaRRD5.2, had a CI of 4.33 cM; and the third, MetaRRD5.3, had a CI of 21.95 cM. The average CI of the individual QTL intervals from all studies on LG 5 was 26.54 cM. On LG 6, the meta-QTL, MetaRRD6.1, had a CI of 9.81 cM, and the second, MetaRRD6.2, had a CI of 8.81 cM. The individual QTL interval from previous studies had an average CI of 35 cM on LG 6, which was larger than the two meta-QTL combined. MetaRRD QTL positions and intervals are presented in Table 6.

MetaRRD5.1 and MetaRRD5.3 primarily result from two QTL each. The remaining 22 QTL in LG 5 support MetaRRD5.2. MetaRRD6.2 is mostly supported by a QTL from the diploid population for severity in the year 2021 and a QTL from T7.20 × SE for the severity of RRD in 2021. The remaining seven QTL correspond to MetaRRD6.1.

### 3.2. Candidate Gene Search

All markers found within our MetaRRD intervals were extracted, and the maximum and minimum marker windows in the base pairs (bp) were used to set the intervals for our gene search. The total number of genes in each interval can be found in Table 7. We searched for genes that could be involved in reduced susceptibility to RRV. Initially, we looked for genes related to antiviral mechanisms, genes similar to ISG15, or genes in pathways involved in antiviral properties. ISG15 activates a ubiquitin-like post-translational modification that regulates de novo synthesized viral or cellular proteins [38]. One gene, RC5G0139400, in the MetaRRD5.2 interval encoded a nucleoporin protein, Ndc1-Nup. Ndc1 controls the number of nuclear pore complexes, which determines the bidirectional movement of macromolecules across the nuclear envelope [39]. A study in Arabidopsis involving mutations in a similar gene encoding for Nuc160 found that mutants showed reduced basal disease resistance [40].

In addition to genes involved in antiviral mechanisms, we searched for possible genes related to disease resistance. Homologs for genes that encode proteins that contain a nucleotide-binding site and leucine-rich repeats (NBS-LRRs) and proteins with a central nucleotide-binding domain, known as NB-ARCs, were found in our meta-QTL intervals. In LG 1, we found 31 hits for NB-ARCs and 30 hits for NBS-LRRs that co-localized to MetaRRD1.1. In LG 3, we found within the MetaRRD3.1 interval one NB-ARC gene and five NBS-LRR genes. For MetaRRD5.1, we found six and four NB-ARC and NBS-LRR genes, respectively. Co-localized with MetaRRD5.2 were four NB-ARC and seven NBS-LRR genes. In the MetaRRD5.3 region, there were 20 NB-ARCs and 4 NBS-LRRs. Finally, in LG 6, MetaRRD6.1 had three NB-ARC and sixteen NBS-LRR genes, while MetaRRD6.2 had six and fourteen hits for NB-ARC and NBS-LRR genes, respectively (Table 7).

Genes involved in viral RNA translation and synthesis were also found within our meta-QTL regions. As these are host factors that contribute to plant viral susceptibility, they may be suitable targets for further investigation. On LG 1, 12 genes involved in viral RNA synthesis or translation were found in the interval of MetaRRD1.1. In LG 3, MetaRRD3.1 had five genes. For LG 5, MetaRRD5.1 had four genes involved in this activity, MetaRRD5.2 had six genes, and MetaRRD5.3 had fifteen. In LG 6, MetaRRD6.1 and MetaRRD6.2 had two and three genes related to viral RNA synthesis or translation, respectively (Table 5). The majority of these genes involve ribosomal proteins (PRs). PRs have been reported to interact and be hijacked by viruses. In the case of the tobacco etch virus (TEV), ribosomal proteins are believed to be directly recruited to enhance translation via base-pairing sequences between the 5′UTR and 18S RNA [41].

We searched for general plant defense responses as well. In the interval of MetaRRD1.1 and 5.3, we found Bet_v_1/MLP-like genes. Bet_v_1 belongs to a family of pathogenesis-related proteins (PR-10) whose expression is induced by pathogen infection, wounding, or abiotic stress [42]. Major latex proteins (MLPs) are the second largest family in the Bet_v_1 protein superfamily and are similarly expressed when invaded by pathogens, helping to defend the plant through innate immunity and acquired resistance signals [43,44]. In MetaRRD3.1 and 5.3, two and one hits, respectively, was observed for another gene related to defense response responsible for the mildew resistance locus O (MLO)-like protein. Originally found in barley conveying fungal powdery mildew resistance, MLO genes are regulated by biotic and abiotic factors, such as fungus and leaf wounding [45]. Resistance due to MLO occurs at the penetration stage through papillae formation and late-acting mesophyll cell death [46]. The remaining eight hits in MetaRRD5.3 were the ankyrin repeat family protein, ACD6, responsible for accelerated cell death with the defense signal salicylic acid [47]. A list of candidate genes by meta-QTL can be found in Supplementary Tables S6–S12.

## 4. Discussion

RRD is the most important viral disease of roses in the USA [2], causing substantial losses in the landscape rose industry. Due to this cost, increasing consideration is being invested into understanding the genetic factors that govern RRD resistance. QTL studies have been used to study resistance to emaraviruses in plants [11,12,13]. This meta-analysis used the two studies performed on roses so far [6,15]. The objectives of this study were to understand the relationship between RRD-resistant QTL in diploid and tetraploid germplasms, better define RRD-resistant QTL intervals, and search for candidate genes in RRD meta-QTL intervals. For this, we looked at two studies that captured one multi-parental diploid and two interconnected bi-parental tetraploid populations across three years. We also remapped the populations from the studies to produce marker maps where the order of markers and genetic distance estimations were improved using the physical position of markers and a multi-point approach [27].

The meta-analysis was conducted on LGs that contained QTL from both diploid [6] and tetraploid [15] populations (LGs 1, 3, 5, and 6). One meta-QTL was identified on LG 1 (MetaRRD1.1), which was roughly a third of the size of the average CI of the QTL from the original studies. All QTL from both diploid and tetraploid populations co-localized with MetaRRD1.1. One meta-QTL was identified on LG 3 (MetaRRD3.1). Three meta-QTL were discovered on LG 5; MetaRRD5.1, MetaRRD5.2, and MetaRRD5.3. All QTL on LG 5 from both studies co-localized with MetaRRD5.2, except for two QTL for severity from the T7.20 × SE population, contributing to MetaRRD5.1, as well as qRRD_TX2WSE_ch5_2021 from the diploid study and qRRV.BE × MG-ch5 from the tetraploid study, contributing to MetaRRD5.3. Thus, our meta-analysis suggests the existence of additional QTL on LG 5 (MetaRRD5.1 and 5.3), which was not evident from the results of separate analyses. However, MetaRRD5.2 was particularly interesting as it was supported by most of the QTL (22) in LG 5. For LG 6, MetaRRD6.1 and MetaRRD6.2 were discovered. The individual QTL from previous studies had an average CI larger than the two meta-QTL combined. As in the case of LG 5, our meta-analysis suggests the presence of two QTL on LG 6, a result that was not evident from the results of separate studies. More research to validate this additional QTL is needed.

When searching for candidate genes, researchers must choose from many possible factors that may be influencing the phenotype of interest [48]. Little research has been performed in the Rosaceae family related to R genes for viral resistance. The discovery of resistance genes in apricots against the plum pox virus [49] is one example within the family. Work has also been undertaken in the distantly related blackcurrant, where they found resistance genes for blackcurrant reversion virus resistance [50]. The results of our meta-analysis provide us with more focused regions of interest for a search of the candidate genes underlying RRD-resistant QTL. The genes that we focused on in our search included genes encoding NBS-LRRs, NB-ARCs, genes associated with antiviral mechanisms, and general defense response. All were identified in the intervals of interest, however, to varying degrees. Genes related to anti-viral mechanisms co-localized to MetaRRD5.2, while homologs of genes encoding disease-resistant proteins with NBS-LRR and NB-ARC domains had numerous hits on all meta-QTL. Other interesting genes found within the meta-QTL regions were those involved in viral RNA translation or synthesis, as well as general defense response genes. Based on the original studies, LG 5 is a major factor in RRD-reduced susceptibility as it accounted for a large portion of the phenotypic variation. The average LG 5 QTL confidence interval from these original studies was ~26.5 cM. In this study, we were able to determine a narrower interval of ~4.3 cM representing ~4 Mbp, allowing a more focused candidate gene search in LG 5. It is important to point out that the candidate gene search we performed used the reference *R. chinensis* genome assembly [32]. Thus, additional work is clearly needed to determine the gene content in QTL intervals in our germplasm.

The results presented here and additional work to better define meta-QTL physical intervals will not only help with candidate gene searches but will allow the identification of marker haplotypes to guide the development of more robust marker-based selection tools to track and use a given QTL in a plant breeding context. Work to identify marker haplotypes in linkage disequilibrium (LD) with key QTL is underway.

## Figures and Tables

**Table 1:** Summary of the QTL found in the inter-related diploid rose populations ^a^.

QTL	Trait ^b^	Year	LG	BF ^c^	PVE (%) ^d^	Position (cM) ^e^	Start (cM)	End (cM)
qRRD_TX2WSE_ch1_2019	Severity	2019	1	2.2	13	8	6	28
qRRD_TX2WSE_ch1_overall	Severity	Overall	1	3.1	14	16	4	20
qRRD_TX2WSE_ch3_overall	Severity	Overall	3	2.1	9.1	18	16	26
qRRV_TX2WSE_ch5_overall	Ctval	Overall	5	9.9	16.3	49	34	54
qRRD_TX2WSE_ch5_overall	Severity	Overall	5	26.9	39.1	49	42	52
qRRD_TX2WSE_ch5_2019	Severity	2019	5	27	24.5	50	36	52
qRRD_TX2WSE_ch5_2020	Severity	2020	5	9.8	14.3	70	54	74
qRRD_TX2WSE_ch5_2021	Severity	2021	5	7.9	10.1	46	40	58
qRRD_TX2WSE_ch6_2021	Severity	2021	6	6.9	12.7	29	16	32

^a^ Table adapted from [6]; ^b^ trait type either for disease severity or virus PCR cycle threshold values (Ctval); ^c^ Bayesian factor—2LnBF; ^d^ percentage of phenotypic variation explained; ^e^ peak position of QTL followed by start and end of the QTL range.

**Table 2:** Summary of QTL found in the independent tetraploid rose populations ^a^.

Population	QTL	Trait ^b^	LG	LOP ^c^	PVE (%) ^d^	Position (cM) ^e^	Start (cM)	End (cM)
SW × BE	qRRD.SW × BE-ch5	Severity	5	6.15	20	35.08	21.01	45.07
	qRRD.SW × BE-ch6	Severity	6	5.63	24	16.01	14.27	33.64
	qRRV.SW × BE-ch5	Ctval	5	3.89	18	26.15	22.03	61.23
BE × MG	qRRD.BE × MG-ch5	Severity	5	6.31	40	17.1	15.08	26.23
	qRRD.BE × MG-ch7	Severity	7	4.15	14	15.03	11.06	29.06
	qRRV.BE × MG-ch5	Ctval	5	3.35	22	36.26	6.19	44.14

^a^ Table adapted from [15]; ^b^ trait type either for disease severity or virus PCR cycle threshold values (Ctval); ^c^ LOP calculated as –log (*p*-value) of the QTL peak; ^d^ percentage of phenotypic variation explained; ^e^ peak position of QTL followed by start and end of the QTL range.

**Table 3:** Summary of QTL found in the joint tetraploid diaQTL analysis ^a^.

QTL	Trait ^b^	Year ^c^	LG	−∆DIC ^d^	PVE (%) ^e^	Position (cM) ^f^	Start (cM)	End (cM)
qRRD-SWBEMG-ch5	Severity	2021	5	−40.81	15	20.31	17.27	26.21
qRRD-SWBEMG-ch6	Severity	2021	6	−21.38	14	10.77	7.65	75.54
qRRD-SWBEMG-ch5	Severity	2021b	5	−48.5	11	20.6	16.46	26.21
qRRD-SWBEMG-ch6	Severity	2021b	6	−19.69	7	13.42	9.81	69.59
qRRD-SWBEMG-ch5	Severity	Overall	5	−49.98	1	20.6	16.46	26.21
qRRD-SWBEMG-ch6	Severity	Overall	6	−23.51	8	12.79	7.52	56.61
qRRV-SWBEMG-ch5	Ctval	Overall	5	−32.81	1	20.37	whole	whole
qRRV-SWBEMG-ch1	Ctval	Overall	1	−18.45	7	3.12	whole	whole

^a^ Table adapted from [15]; ^b^ trait type either for disease severity or virus PCR cycle threshold values (Ctval); ^c^ two sets of observations were taken in 2021, once earlier in the year 2021a (September) and once later in the year 2021b (November); ^d^ deviance information criterion; ^e^ percentage of phenotypic variation explained; ^f^ peak position of QTL followed by start and end of the QTL range.

**Table 4:** Summary of QTL found in the remapped diploid rose populations.

Population	QTL	Trait ^a^	LG	LOP ^b^	PVE (%) ^c^	Position (cM) ^d^	Start (cM)	End (cM)
T7.20 × SE	qRRV_T7.20 × SE_ch3_remap	Ctval	3	4.19	14	9.31	0	35.3
	qRRV_T7.20 × SE_ch5_remap	Ctval	5	15.65	63	28.47	14.23	32.11
	qRRD_T7.20 × SE_ch5_2019_remap	Severity	5	5.91	39	11.25	7.63	32.11
	qRRD_T7.20 × SE_ch5_overall_remap	Severity	5	5.44	36	11.25	7.63	32.11
	qRRD_T7.20 × SE_ch5_2021_remap	Severity	5	4.16	26	29.02	19.27	38.41
	qRRD_T7.20 × SE_ch6_2021_remap	Severity	6	3.42	18	25.43	12.02	28.48
T7.30 × SE	qRRV_T7.30 × SE_chr_remap	Ctval	5	7.84	55	16.33	6.35	28.24
	qRRD_T7.30 × SE_chr5_2020_remap	Severity	5	3.46	30	16.33	8.2	52.12
	qRRD_T7.30 × SE_chr5_overall_remap	Severity	5	3.22	29	16.33	0	51.53

^a^ Trait type either for disease severity or virus PCR cycle threshold values (Ctval); ^b^ LOP calculated as −log (*p*-value) of the QTL peak; ^c^ percentage of phenotypic variation explained; ^d^ peak position of QTL followed by start and end of the QTL range.

**Table 5:** Summary of QTL found in the remapped tetraploid rose populations.

Population	QTL	Trait ^a^	LG	LOP ^b^	PVE (%) ^c^	Position (cM) ^d^	Start (cM)	End (cM)
SW × BE	qRRD_SW × BE_ch5_2020_remap	Severity	5	4.59	20	51.32	22.84	111.23
	qRRD_SW × BE_ch5_2021b_remap	Severity	5	5.72	24	43.39	17.41	50.02
	qRRD_SW × BE_ch6_2021b_remap	Severity	6	6.11	24	14.12	6.97	25.27
	qRRD_SW × BE_ch5_2021_remap	Severity	5	4.39	19	43.39	16.14	57.14
	qRRD_SW × BE_ch6_2021_remap	Severity	6	5.73	25	14.12	5.31	25.27
	qRRD_SW × BE_ch5_overall_remap	Severity	5	6.25	19	43.39	19.77	58.14
	qRRD_SW × BE_ch6_overall_remap	Severity	6	5.32	27	12.96	5.31	27.61
	qRRV_SW × BE_ch3_remap	Ctval	3	3.52	25	32.36	0	106
BE × MG	qRRD_BE × MG_ch5_2021b_remap	Severity	5	3.65	32	28.33	17.65	57.05
	qRRD_BE × MG_ch5_2021_remap	Severity	5	4.16	38	28.33	17.65	59.04
	qRRD_BE × MG_ch5_overall_remap	Severity	5	6.53	39	39	17.65	48.27
	qRRD_BE × MG_ch7_overall_remap	Severity	7	3.99	15	11.04	7.07	54.6
	qRRV_BE × MG_chr5_remap	Ctval	5	4.42	33	28.33	9.18	61.06

^a^ Trait type either for disease severity or virus PCR cycle threshold values (Ctval); ^b^ LOP calculated as −log (*p*-value) of the QTL peak; ^c^ percentage of phenotypic variation explained; ^d^ peak position of QTL followed by start and end of the QTL range.

**Table 6:** Meta-QTL genomic and genetic positions and intervals for RRD resistance in roses.

MetaQTL ^a^	Position (cM)	Start (cM)	End (cM)	Start (Mbp) ^b^	End (Mbp) ^b^
MetaRRD1.1	20.92	15.655	26.185	1.585	12.455
MetaRRD3.1	40.87	37.88	43.86	20.58	24.525
MetaRRD5.1	11.91	3.225	20.595	1.235	6.005
MetaRRD5.2	34.8	32.635	36.965	9.22	13.05
MetaRRD5.3	64.09	53.115	75.065	19.70	40.51
MetaRRD6.1	15.74	10.835	20.645	4.18	12.175
MetaRRD6.2	37.7	33.295	42.105	19.50	31.67

^a^ MetaQTL name coded as MetaRRD followed by the LG and then QTL number. ^b^ Start and stop in Mbp based on the 5000 base pair (bp) window of the closest marker.

**Table 7:** Candidate genes in the MetaQTL regions for RRD resistance in roses.

Meta-QTL	Number of Genes in an Interval					
	Antiviral Mechanism ^a^	NBS-LRR ^b^	NB-ARC ^c^	Viral mRNA ^d^	Defense Response ^e^	Total ^f^
MetaRRD1.1	0	30	31	12	2	886
MetaRRD3.1	0	5	1	5	3	381
MetaRRD5.1	0	4	6	4	0	630
MetaRRD5.2	1	7	4	6	0	437
MetaRRD5.3	0	4	20	15	12	1700
MetaRRD6.1	0	16	3	2	0	713
MetaRRD6.2	0	14	6	3	0	691

^a^ Number of genes associated with antiviral mechanisms; ^b^ number of genes in the interval with NBS-LRR domain-containing disease-resistant proteins; ^c^ number of genes with NB-ARC domain-containing disease-resistant proteins; ^d^ number of genes related to viral mRNA synthesis/translation; ^e^ genes related to general defense response; ^f^ total number of annotated genes in the meta-QTL interval.

## Data Availability

The data presented in this study are available on request from the corresponding author.

## Supplementary Materials

The following supporting information can be downloaded at: https://www.mdpi.com/article/10.3390/pathogens12040575/s1, Figure S1: Consensus map of all studies; Table S1: Linkage map summary for BE × MG; Table S2: Linkage map summary for SW × BE; Table S3: Linkage map summary for J14.3 × PH; Table S4: Linkage map summary for T7.20 × SE; Table S5: Linkage map summary for T7.30 × SE; Table S6: Candidate genes co-localized to MetaRRD1.1; Table S7: Candidate genes co-localized to MetaRRD3.1; Table S8: Candidate genes co-localized to MetaRRD5.1; Table S9: Candidate genes co-localized to MetaRRD5.3; Table S10: Candidate genes co-localized to MetaRRD5.3; Table S11: Candidate genes co-localized to MetaRRD6.1; Table S12: Candidate genes co-localized to MetaRRD6.2.
