# Supplementary material for: Meta-Analysis of Rose Rosette Disease-Resistant Quantitative Trait Loci and a Search for Candidate Genes
Source: Pathogens. 2023 Apr 8;12(4):575. doi: 10.3390/pathogens12040575 (PMC10146096; doi:10.3390/pathogens12040575)
Supplement: Supplementary file 1 [file pathogens-12-00575-s001.zip › Figure_S1.pdf]

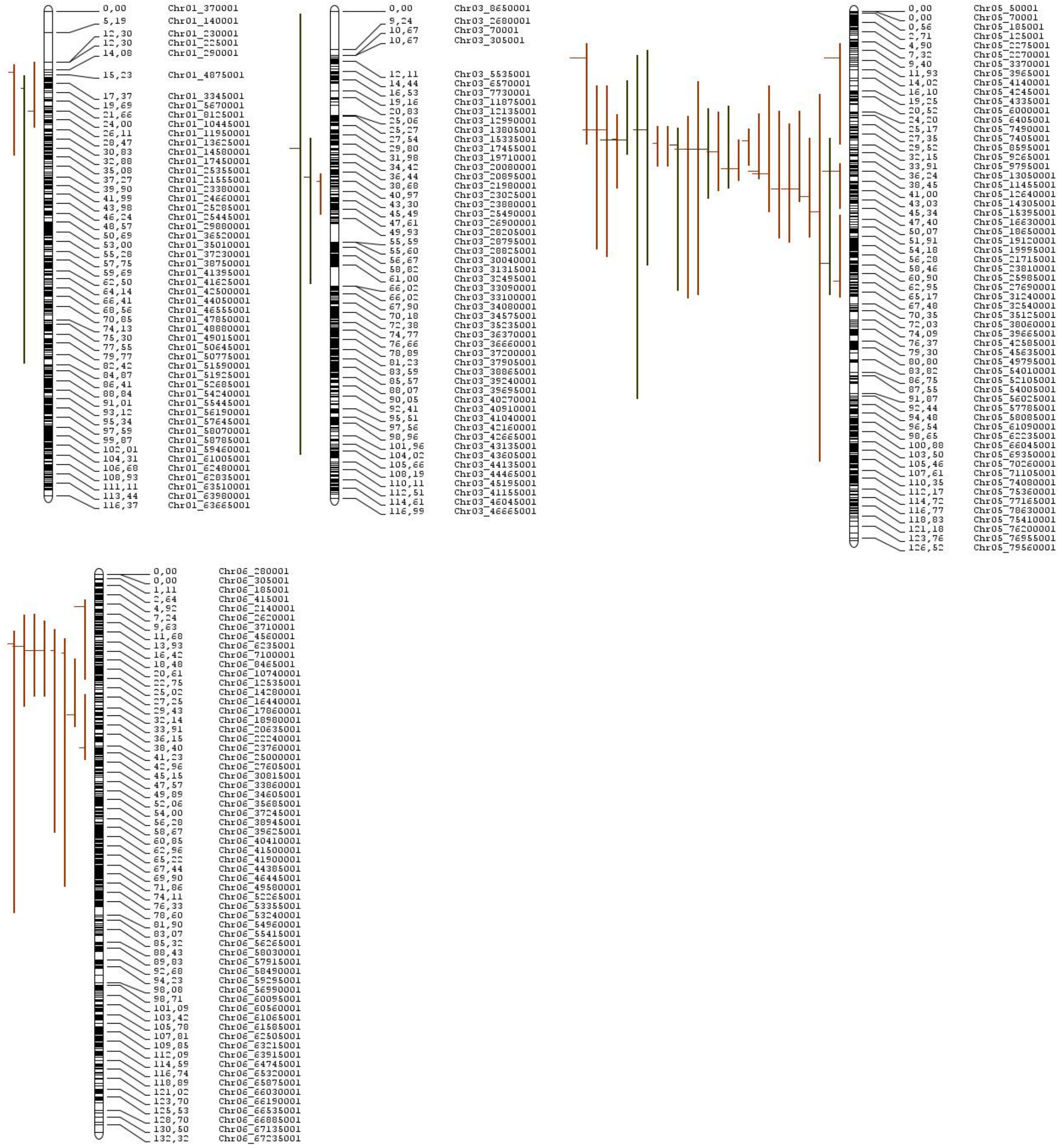

Figure S1. Consensus map of all data sets. Individual QTL to the left of the LG, and marker names with their position in cM to the right.
